# Supplementary material for: Return to theatre for post-tonsillectomy haemorrhage in children has not fallen with increased use of plasma ablation tonsillectomy: a retrospective analysis of 359,241 tonsillectomies in 15 years of United Kingdom Hospital Episode Statistics
Source: PLoS One. 2025 Jul 28;20(7):e0328251. doi: 10.1371/journal.pone.0328251 (PMC12303344; doi:10.1371/journal.pone.0328251)
Supplement: Attachment — Submitted filename: PONE-D-25-08145.pdf [file pone.0328251.s002.pdf]

PLOS ONE

Return to theatre for post-tonsillectomy haemorrhage in children has not fallen with increased use of plasma ablation tonsillectomy: a retrospective analysis of 359,241 tonsillectomies in 15 years of United Kingdom Hospital Episode Statistics

--Manuscript Draft--

**Manuscript Number:**	PONE-D-25-08145
**Article Type:**	Research Article
**Full Title:**	Return to theatre for post-tonsillectomy haemorrhage in children has not fallen with increased use of plasma ablation tonsillectomy: a retrospective analysis of 359,241 tonsillectomies in 15 years of United Kingdom Hospital Episode Statistics
**Short Title:**	Return to theatre rates for postoperative haemorrhage with increasing plasma ablation tonsillectomy
**Corresponding Author:**	Jeremy Reid<br>University of Birmingham<br>Birmingham, UNITED KINGDOM OF GREAT BRITAIN AND NORTHERN IRELAND
**Keywords:**	plasma ablation tonsillectomy; post-tonsillectomy haemorrhage; paediatric tonsillectomy; Hospital Episode Statistics; Patient safety
**Abstract:**	Introduction: Plasma ablation tonsillectomy has rapidly increased in popularity and is now the most popular technique in children. This study aims to evaluate the impact of plasma ablation tonsillectomy on the incidence of post-operative haemorrhage requiring surgical intervention in children, a complication affecting patient safety and healthcare resource utilisation. Methods: 15 years of Hospital Episode Statistics for children 14 years or under, capturing all tonsillectomies in NHS England hospitals was analysed. The proportion performed by plasma ablation and rate of surgical intervention for post-tonsillectomy haemorrhage were calculated. Pearson's Correlation Coefficient was used to statistically analyse the relationship. Results: Data from 359,241 tonsillectomies was analysed. The proportion of tonsillectomies performed with plasma ablation has grown yearly from 7% in 2009/10 to 47% in 2023/24. A change in trend in the rate of return to theatre for haemorrhage control was not identified across the study period. (Pearson Correlation Coefficient -0.15, p=0.59) Conclusion: These findings do not support a superior safety profile of plasma ablation tonsillectomy with regard to post-operative haemorrhage. However, due to dataset limitations it was not possible to analyse intracapsular and extracapsular procedures independently. There remains a need for continued evaluation of tonsillectomy techniques to inform optimal surgical practice.
**Order of Authors:**	Jeremy Reid
	Thomas Beech
	Jameel Muzaffar
**Additional Information:**	
**Question**	**Response**
**Financial Disclosure**<br><br>Enter a financial disclosure statement that describes the sources of funding for the work included in this submission. Review the submission guidelines for detailed requirements. View published research articles from *PLOS ONE* for specific examples.	The author(s) received no specific funding for this work.

Powered by Editorial Manager® and ProduXion Manager® from Aries Systems Corporation

This statement is required for submission and **will appear in the published article** if the submission is accepted. Please make sure it is accurate.<br><br>**Funded studies**<br>Enter a statement with the following details:<br>• Initials of the authors who received each award<br>• Grant numbers awarded to each author<br>• The full name of each funder<br>• URL of each funder website<br>• Did the sponsors or funders play any role in the study design, data collection and analysis, decision to publish, or preparation of the manuscript?<br><br>Did you receive funding for this work?	
**Competing Interests**<br><br>Use the instructions below to enter a competing interest statement for this submission. On behalf of all authors, disclose any competing interests that could be perceived to bias this work—acknowledging all financial support and any other relevant financial or non-financial competing interests.<br><br>This statement is required for submission and **will appear in the published article** if the submission is accepted. Please make sure it is accurate and that any funding sources listed in your Funding Information later in the submission form are also declared in your Financial Disclosure statement.<br><br>View published research articles from *PLOS ONE* for specific examples.	The authors have declared that no competing interests exist

**NO authors have competing interests**<br><br>Enter: *The authors have declared that no competing interests exist.*<br><br>**Authors with competing interests**<br><br>Enter competing interest details beginning with this statement:<br><br>*I have read the journal's policy and the authors of this manuscript have the following competing interests: [insert competing interests here]*<br><br>* typeset	
**Ethics Statement**<br><br>Enter an ethics statement for this submission. This statement is required if the study involved:<br><br>• Human participants<br>• Human specimens or tissue<br>• Vertebrate animals or cephalopods<br>• Vertebrate embryos or tissues<br>• Field research<br><br>Write "N/A" if the submission does not require an ethics statement.<br><br>General guidance is provided below. Consult the submission guidelines for detailed instructions. **Make sure that all information entered here is included in the Methods section of the manuscript.**	N/A

Powered by Editorial Manager® and ProduXion Manager® from Aries Systems Corporation

**Format for specific study types**<br><br>**Human Subject Research (involving human participants and/or tissue)**<br>• Give the name of the institutional review board or ethics committee that approved the study<br>• Include the approval number and/or a statement indicating approval of this research<br>• Indicate the form of consent obtained (written/oral) or the reason that consent was not obtained (e.g. the data were analyzed anonymously)<br><br>**Animal Research (involving vertebrate animals, embryos or tissues)**<br>• Provide the name of the Institutional Animal Care and Use Committee (IACUC) or other relevant ethics board that reviewed the study protocol, and indicate whether they approved this research or granted a formal waiver of ethical approval<br>• Include an approval number if one was obtained<br>• If the study involved *non-human primates*, add *additional details* about animal welfare and steps taken to ameliorate suffering<br>• If anesthesia, euthanasia, or any kind of animal sacrifice is part of the study, include briefly which substances and/or methods were applied<br><br>**Field Research**<br><br>Include the following details if this study involves the collection of plant, animal, or other materials from a natural setting:<br>• Field permit number<br>• Name of the institution or relevant body that granted permission	
**Data Availability**<br><br>Authors are required to make all data underlying the findings described fully available, without restriction, and from the time of publication. PLOS allows rare exceptions to address legal and ethical concerns. See the PLOS Data Policy and FAQ for detailed information.	Yes - all data are fully available without restriction

A Data Availability Statement describing where the data can be found is required at submission. Your answers to this question constitute the Data Availability Statement and **will be published in the article**, if accepted.<br><br>**Important:** Stating 'data available on request from the author' is not sufficient. If your data are only available upon request, select 'No' for the first question and explain your exceptional situation in the text box.<br><br>Do the authors confirm that all data underlying the findings described in their manuscript are fully available without restriction?	
**Describe where the data may be found in full sentences. If you are copying our sample text, replace any instances of XXX with the appropriate details.**<br><br>• If the data are **held or will be held in a public repository**, include URLs, accession numbers or DOIs. If this information will only be available after acceptance, indicate this by ticking the box below. For example: *All XXX files are available from the XXX database (accession number(s) XXX, XXX.*).<br>• If the data are all contained **within the manuscript and/or Supporting Information files**, enter the following: *All relevant data are within the manuscript and its Supporting Information files.*<br>• If neither of these applies but you are able to provide **details of access elsewhere**, with or without limitations, please do so. For example:<br><br>*Data cannot be shared publicly because of [XXX]. Data are available from the XXX Institutional Data Access / Ethics Committee (contact via XXX) for researchers who meet the criteria for access to confidential data.*<br><br>*The data underlying the results presented in the study are available from (include the name of the third party*	All data available from NHS Digital. Hospital Admitted Patient Care Activity. Available: https://digital.nhs.uk/data-and-information/publications/statistical/hospital-admitted-patient-care-activity

and contact information or URL).<br>• This text is appropriate if the data are owned by a third party and authors do not have permission to share the data.<br><br>* typeset	
Additional data availability information:	

Powered by Editorial Manager® and ProduXion Manager® from Aries Systems Corporation

Manuscript

Click here to access/download;Manuscript;Write up HES coblation tonsillectomy PLOS ver2.docx

Full title: (242 characters): **Return to theatre for post-tonsillectomy haemorrhage in children has not fallen with increased use of plasma ablation tonsillectomy: a retrospective analysis of 359,241 tonsillectomies in 15 years of United Kingdom Hospital Episode Statistics**

Short title: (99 characters): **Return to theatre rates for postoperative haemorrhage with increasing plasma ablation tonsillectomy**

Authors: Jeremy Reid*[1,2] (ORCiD 0000-0003-4399-6242)
Thomas Beech[2] (ORCiD 0000-0002-6110-5995)
Jameel Muzaffar[2] (ORCiD 0000-0003-3065-0269)

Affiliation: [1]College of Medicine and Health
University of Birmingham
Edgbaston
Birmingham, B15 2TT
United Kingdom

[2]Department of Ear, Nose and Throat Surgery
University Hospitals Birmingham NHS Foundation Trust
Mindelsohn Way
Edgbaston
Birmingham, B15 2GW
United Kingdom

*Corresponding author: jpr128@student.bham.ac.uk

Acknowledgements: Nil

Funding: Nil

Authorship statement: JR study concept, design, data collection, analysis, article writing. TB data analysis and article writing. JM data analysis and article writing.

Conflicts of interest: No authors have any conflicts of interest to declare.

ABSTRACT: (199 words)

Introduction: Plasma ablation tonsillectomy has rapidly increased in popularity and is now the most popular technique in children. This study aims to evaluate the impact of plasma ablation tonsillectomy on the incidence of post-operative haemorrhage requiring surgical intervention in children, a complication affecting patient safety and healthcare resource utilisation. **Methods**: 15 years of Hospital Episode Statistics for children 14 years or under, capturing all tonsillectomies in NHS England hospitals was analysed. The proportion performed by plasma ablation and rate of surgical intervention for post-tonsillectomy haemorrhage were calculated. Pearson's Correlation Coefficient was used to statistically analyse the relationship. **Results**: Data from 359,241 tonsillectomies was analysed. The proportion of tonsillectomies performed with plasma ablation has grown yearly from 7% in 2009/10 to 47% in 2023/24. A change in trend in the rate of return to theatre for haemorrhage control was not identified across the study period. (Pearson Correlation Coefficient -0.15, p=0.59) **Conclusion**: These findings do not support a superior safety profile of plasma ablation tonsillectomy with regard to post-operative haemorrhage. However, due to dataset limitations it was not possible to analyse intracapsular and extracapsular procedures independently. There remains a need for continued evaluation of tonsillectomy techniques to inform optimal surgical practice.

Key words: plasma ablation tonsillectomy, post-tonsillectomy haemorrhage, paediatric tonsillectomy, Hospital Episode Statistics, patient safety

Key points:

- Tonsil surgery is very common with post-operative bleeding from the tonsillar bed being a potentially life threatening complication
- Current evidence points to a lower incidence of post-tonsillectomy haemorrhage with intracapsular plasma ablation tonsillectomy compared to extracapsular techniques
- This analysis of 15 years of national level paediatric tonsillectomy data has not identified a correlation between the rising popularity of plasma ablation technique tonsillectomy and falling return to theatre rates for post-tonsillectomy haemorrhage
- Differentiating between intracapsular and extracapsular plasma ablation technique in this dataset has not been possible which limits the certainty of the conclusions

Introduction

Background and Rationale

Tonsillectomy is one of the most commonly performed operations with approximately 40-50,000 procedures performed annually in the UK National Health Service with more than half of all cases performed in children. Common indications are recurrent tonsillitis and paediatric obstructive sleep apnoea. Morbidity associated with tonsillectomy includes post-operative haemorrhage (primary within 24 hours, or secondary from 24 hours to 28 days post-operatively[1]) from the tonsillar bed which can potentially be profuse, leading to airway and circulatory crisis. Minor bleeding is not

uncommon, with one study reporting a 44% incidence of 'blood in saliva' or greater.[2] Conservative management may be appropriate for minor bleeds but post-tonsillectomy haemorrhage may require an emergency return to theatre for haemorrhage control, especially in younger children with lower circulating blood volume.

Techniques for tonsillectomy have been widely debated. Factors affecting choice of surgical technique include patient outcomes, cost, duration of procedure, environmental impact and surgeon preference. 'Plasma ablation' (Coblation®, Smith&Nephew) is a technology that utilises a single use 'wand' to create a plasma field. This allows precise disintegration of tissues with significantly lower thermal exposure than techniques such as bipolar, monopolar or laser dissection.[1,3] Plasma ablation tonsillectomy can be performed through extracapsular dissection (the same dissection plane as traditional cold steel or bipolar technique) or the more recently developed intracapsular ablation of tonsillar tissue which avoids disruption to the tonsillar capsule. Preservation of the capsule is thought to minimise trauma to underlying nerves and larger vessels resulting in a reduction in postoperative pain and reduced incidence of post-tonsillectomy haemorrhage.[4]

Tonsillectomy has been shown in the recent NAtional Trial of Tonsillectomy IN Adults (NATTINA) to be a clinically effective and cost-effective way of managing recurrent acute tonsillitis.[5] The National Prospective Tonsillectomy Audit (NPTA) was performed in 2003, with results showing primary bleed rates across all techniques of 0.6% and secondary bleed rates of 3%.[6] Total tonsillar haemorrhage rates reported by the NPTA were most favourable for cold steel at 1.7%, with bipolar diathermy forceps 4.6% and extracapsular plasma ablation 4.6%. Return to theatre rates were 0.8% for cold steel, 1.0% for bipolar diathermy forceps and 1.8% for extracapsular plasma ablation.[6] A subsequent Cochrane systematic review reported a non-statistically significant elevated secondary bleeding risk with extracapsular plasma ablation with absolute risk of 5% in the plasma ablation group vs 3.6% in the control group.[7]

Since these publications the intracapsular plasma ablation tonsillectomy technique has grown in popularity with evidence suggesting that compared to extracapsular plasma ablation it results in reduced late post-operative pain and a much lower incidence of post-tonsillectomy haemorrhage.[8] A single surgeon case series of 1,257 paediatric patients who underwent intracapsular plasma ablation reports a post-tonsillectomy haemorrhage rate of 0.5% with 0% requiring return to theatre.[9] A retrospective cohort study of four centres exclusively performing paediatric plasma ablation tonsillectomy reports a return to theatre rate for primary haemorrhage of 0.1% and of secondary haemorrhage of 0.2%.[10] Due to such promising data, intracapsular plasma ablation has been promoted in national guidance and it has been steadily growing in popularity in United Kingdom paediatric tonsillectomy practice.[11]

Hospital Episode Statistics (HES) refer to routinely collected data for all UK National Health Service (NHS) care episodes in England, including elective and emergency care, capturing demographic information, diagnoses and procedures. This anonymised data is published annually and made publicly available by NHS Digital.

The aim of this study was to use HES data to assess UK trends in paediatric tonsillectomy practice and to assess if the reported reduction in post-tonsillectomy haemorrhage rate is reflected in national level data.

Objectives

- To assess the trend in the use of plasma ablation tonsillectomy in the UK over the last 15 years.

- To evaluate the association between the increased use of plasma ablation tonsillectomy and the rate of return to theatre for post-tonsillectomy haemorrhage.
- To evaluate the association between increased use of plasma ablation tonsillectomy and rates of surgery to remove remnant tonsils.

Hypothesis

- As the proportion of tonsillectomies performed by plasma ablation increases the rate of return to theatre for arrest of post-tonsillectomy haemorrhage will decrease.
- As the proportion of tonsillectomies performed by plasma ablation increases the rate of excision of tonsillar remnants will increase.

Methods

Study Design & Setting

This study employed a historical observational design utilising publicly available routinely collected data over a sequential 15-year period from the United Kingdom National Health Service (NHS). This approach allowed for the examination of long-term trends and outcomes associated with different tonsillectomy techniques providing insights into surgical practice and patient outcomes. We have reported our findings in accordance with Strengthening the Reporting of Observational Studies in Epidemiology (STROBE) guidelines.

Participants

Hospital Episode Statistics (Admitted Patient Care Dataset) was accessed providing fifteen sequential years of data capturing all NHS England hospital care episodes including day case surgery from years 2009/10 to 2023/24.[12] Only data for patients 14 years or under was extracted for analysis in the study. 14 years of age was chosen as datasets from 2011/12 and earlier grouped 15- and 16-year-olds together with adults rather than children. Data extracted included total number of tonsillectomy procedures by technique and complications of surgical arrest of postoperative bleeding from tonsillar bed and excision of remnant tonsil.

Ethical Considerations

HES data does not include patient identifiable information and no additional data collection was performed. Therefore no significant ethical concerns were identified and formal ethical approval was not sought.

Variables

Data detailing annual tonsillectomy procedures performed across several techniques, including dissection, guillotine, laser, excision not elsewhere classified (NEC), and plasma ablation as available from HES data.

Bias

Given the historical nature of the study and the reliance on existing records for data collection, several sources of bias were considered:

Selection Bias: The study population was derived from a dataset that included all patients undergoing tonsillectomy within the specified time frame. This approach minimises the risk of selection bias related to the inclusion criteria, however varying geographical preferences for surgical technique could introduce bias. Specifically, the decision to use plasma ablation versus other tonsillectomy techniques may be influenced by institutional practices or surgeon preferences/case volume, which are not accounted for in our dataset.

Information Bias: Inaccuracies in clinical coding could lead to misclassification bias, for example revision surgery for tonsillar remnant may be incorrectly recorded as a primary procedure by whichever technique was used.

Confounding Bias: The absence of individual patient-level data, such as age, sex, and indication for tonsillectomy, limits our ability to adjust for possible confounding factors. These unmeasured confounders could influence both the choice of tonsillectomy technique and the risk of postoperative haemorrhage.

Study Size

The size of our study cohort was determined by the availability of recorded data within the specified timeframe.

Data handling and statistical methods

The total number of tonsillectomies was calculated by summing figures for OPCS (Office for Population and Censuses Surveys) codes F34.1 (Bilateral dissection tonsillectomy), F34.2 (Bilateral guillotine tonsillectomy), F34.3 (Bilateral laser tonsillectomy), F34.4 (Bilateral excision of tonsil NEC)

and F34.7 (Bilateral coblation tonsillectomy). Percentages were calculated each year for numerators of F34.7 (Bilateral coblation tonsillectomy) and F36.5 (Surgical arrest of postoperative bleeding from tonsillar bed). For the final 2023/24 dataset, in addition to the code F34.7 (Bilateral coblation tonsillectomy, n=8005) additional codes were introduced not found in previous years. These were F35.1 (Bilateral intracapsular tonsillectomy NEC, n=213), F35.2 (Bilateral intracapsular coblation tonsillectomy, n=4080), F35.8 (Other specified intracapsular excision of tonsil, n=17) and F35.9 (Unspecified intracapsular excision of tonsil, n=23). Absolute numbers for non-plasma ablation intracapsular procedures (F35.1, F35.8 and F35.9) are very low and we suspect these represent coding errors for intracapsular plasma ablation procedures as non-plasma ablation intracapsular tonsillectomy does not represent typical practice. Therefore these four codes were summed to represent intracapsular coblation tonsillectomy and these numbers were added to F34.7 to calculate the total number of coblation tonsillectomies that year to allow comparison with datasets from previous years.

To assess the association between the use of plasma ablation tonsillectomy and (a) the rate of return to theatre for post-tonsillectomy haemorrhage and (b) excision of remnant tonsil, Pearson's correlation coefficient was calculated to quantify the strength and direction of association between variables. Statistical analyses were conducted using Python, and a two-tailed p-value <0.05 was considered statistically significant.

Results

359,241 tonsillectomies were included over the fifteen years of study data. Data are summarised in Table 1. Total tonsillectomies fell sharply for the year 2020/2021 before recovering in the subsequent two years. The proportion of tonsillectomies performed with plasma ablation technique hovered

around 7% until 2011/12, after which it has risen steadily year on year up to 47.1% in 2022/23, now representing the single most frequently used technique. (See Figure 1) The rate of return to theatre for arrest of postoperative haemorrhage hovered around 0.7% for the first four years of data before jumping to 1% in 2013/14. It stayed between 0.84% and 1.04% for the next 6 years, before dipping in 2020/21 and 2020/22 before rising again to 0.79% in 2022/23 and 0.85% in 2023/24. (See Figure 2) Pearson's Correlation Coefficient was calculated as -0.15 (p=0.59) indicating no clear correlation between use of plasma ablation technique tonsillectomy and rate of return to theatre for control of post-tonsillectomy haemorrhage. Excision of remnant tonsil (F34.5) rate demonstrated a non-statistically significant moderate upward trend over the study period (Pearson Correlation Coefficient 0.42, p=0.11) with low absolute numbers (range 4-23). (See Figure 3) Data from 2023/24 includes new OPCS codes as explained above and confirms at least 33% of plasma ablation tonsillectomies were performed with intracapsular technique.

**Year**	**Dissection**	**Guillotine**	**Laser**	**Excision NEC**	**Plasma ablation**	**Total Procedures**	**% Plasma ablation**	**% Dissection**	**% Return to theatre**
2009/10	21,854	198	14	2,576	1,985	26,627	7.45%	82.07%	0.79%
2010/11	21,281	120	21	2,302	1,621	25,345	6.40%	83.97%	0.63%
2011/12	20,725	147	41	2,415	1,861	25,189	7.39%	82.28%	0.76%
2012/13	20,862	101	21	2,653	2,016	25,653	7.86%	81.32%	0.71%
2013/14	22,332	120	11	2,884	2,957	28,304	10.45%	78.90%	1.00%
2014/15	22,436	149	12	2,762	3,418	28,777	11.88%	77.97%	1.04%
2015/16	21,218	113	15	2,571	3,845	27,762	13.85%	76.43%	0.93%
2016/17	19,610	99	14	3,034	4,954	27,711	17.88%	70.77%	0.93%
2017/18	18065	91	25	2,165	5,723	26,069	21.93%	69.30%	0.94%
2018/19	16,276	86	32	2,348	7,299	26,041	28.03%	62.50%	0.84%
2019/20	13,558	63	16	2,078	7,514	23,229	32.35%	58.37%	0.93%
2020/21	4,180	52	6	917	3,436	8,591	40.00%	48.66%	0.72%
2021/22	6,874	34	8	1,737	6,285	14,938	42.07%	46.02%	0.65%
2022/23	7,970	27	12	2,220	8,577	18,806	45.61%	42.38%	0.79%
2023/24	11,199	21	18	2,623	12,338*	26,199	47.09%	42.75%	0.85%

Table 1: Annual Tonsillectomy Procedures and Proportions. *Note: The "%" columns for Plasma ablation and Dissection are calculated as the proportion of those techniques relative to the total procedures performed each year. NEC = Not Elsewhere Classified. *Please see 'Data handling and statistical methods'*

Fig 1: Annual number of paediatric tonsillectomies by technique. *The proportion of tonsillectomies performed by plasma ablation technique has risen steadily from 6.4% in 2010/11 to 47.1% in 2023/24.*

Fig 2: Proportion of surgical arrests of post-tonsillectomy haemorrhage and proportion of plasma ablation tonsillectomies over time. *Pearson Correlation Coefficient -0.15, p=0.59.*

Fig 3: Proportion of remnant tonsil excision and plasma ablation tonsillectomies over time. *Pearson Correlation Coefficient 0.42, p=0.11.*

Discussion

This analysis of national routinely collected data has shown that the proportion of paediatric tonsillectomies performed by plasma ablation technique in the United Kingdom has been steadily rising however a fall in the return to theatre rate for post-tonsillectomy haemorrhage rate has not been identified. Indeed, the return to theatre rate was higher in 2023/24 when nearly half of

paediatric tonsillectomies were performed by plasma ablation compared to 2010/11 when only 6% of tonsillectomies were performed by plasma ablation. There was a small drop in return to theatre rate in 2020/21 and 2021/22, which likely represents a predilection for conservative management during the covid-19 pandemic. It is noteworthy that there was also no positive correlation identified suggesting plasma ablation is not an inferior technique with regard to post-tonsillectomy haemorrhage.

A key point of discussion with this analysis is that HES data does not distinguish between extracapsular dissection and intracapsular ablation approaches of using plasma ablation with only a single code for 'Coblation tonsillectomy' (F34.7) for datasets prior to 2023/24. The previously published studies supporting lower rates of post-tonsillectomy haemorrhage with plasma ablation technique are only in support of the intracapsular approach.[9,10] We may therefore not be seeing return to theatre rates fall if the benefits of intracapsular plasma ablation are being masked by a large proportion of extracapsular plasma ablation procedures which are reported to carry an increased bleeding risk.[6,7] For the 2023/24 dataset, as well as F34.7, new codes for intracapsular procedures have been introduced. This follows calls for HES data to provide better granularity on plasma ablation technique.[10,11,13] Unfortunately despite confirming at least 33% of plasma ablation tonsillectomies were performed with intracapsular technique, these do not facilitate confirmation of whether the remaining plasma ablation procedures were intracapsular or extracapsular as F34.7 'Bilateral coblation tonsillectomy' was still included. However, even accounting for a proportion of extracapsular procedures in the plasma ablation group, we are surprised not to identify some degree of trend considering the strength of the previously published data in favour of the intracapsular technique.[9,10]

We suspect most of the procedures represented by F34.7 are intracapsular as the increased bleeding risk for extracapsular technique was reported by the NPTA four years prior to our study period

beginning. We think it unlikely surgeons or industry taught courses would promote what is established to be an inferior technique.[6] Furthermore, for the 2023/24 dataset, the new OPCS codes will be unfamiliar to surgeons and theatre staff and we suspect many will continue to code as they did previously, therefore under-reporting the proportion of intracapsular plasma ablation procedures. We cannot be sure of this however and if a significant proportion of the plasma ablation group is in fact extracapsular then this would affect our conclusions. We would encourage all surgeons to ensure their procedures are accurately coded to allow future HES data to be best utilised for analysing different techniques of plasma ablation tonsillectomy. We would also recommend for OPCS code F34.7 'Bilateral coblation tonsillectomy' to be removed and replaced with 'Bilateral extracapsular coblation tonsillectomy' so in conjunction with the new 2023/24 codes the number of procedures performed by each technique is clear.

It is recognised that optimal use of plasma ablation has a learning curve, and a possible influence on the data is that as the technique grows in popularity there may be more novice operators failing to achieve optimal results.[14] However this would only apply to a small subset of plasma ablation operations and should also be balanced by a corresponding increase in the number of experienced operators performing or supervising tonsillectomies.

Another explanation for the data is that the benefits of plasma ablation technique could be masked if the return to theatre rate for non-plasma ablation techniques such as bipolar dissection has risen. For example if trainees or less experienced surgeons who fail to achieve optimal results prefer traditional techniques this may increase the bleed rate in the data. However, we would expect that while under supervision trainees would adopt and learn the preferred technique of their supervisor. It could also be suggested that more challenging cases with a higher rebleed risk such as those of recurrent quinsy are less likely to be performed with plasma ablation. While for such situations this is probably the case it would only infrequently apply to a paediatric population 14 years and under.

An advantage of this analysis is that we have a very large dataset including all tonsillectomies in NHS England hospitals for children 14 years and under over a sequential fifteen-year period. This gives excellent power to the study for detecting a real change if one is present. Furthermore, the existing evidence base originates from a small number of expert centres for which there may be factors within such a set up that limit external validity. This study uses national level data and therefore represents the heterogeneity of real-world usage of coblation tonsillectomy.

The upward trend in rate of 'Remnant tonsil excision' (F34.5) demonstrated considerable variation and did not reach statistical significance. We suspect the very low absolute numbers partially reflect inaccurate coding of re-excisions as primary procedures instead of F34.5. A lag time of multiple years is to be expected between a primary procedure and a re-excision procedure. We therefore feel this outcome is inadequately assessed by the current study. However, a non-statistically significant moderate upward trend was identified and we recommend that this outcome continues to be monitored.

Conclusion

Despite the theoretical advantages of plasma ablation, this study of a fifteen-year dataset of paediatric tonsillectomies across NHS hospitals in England has not supported its adoption being linked with a reduction in the rate of surgical intervention for post-tonsillectomy haemorrhage. As it has not been possible to separate intracapsular from extracapsular procedures we cannot conclude that intracapsular plasma ablation does not have a lower return to theatre rate, however this reported advantage has not been corroborated in this national level dataset. Considering the enormous case load of tonsil surgery and in view of the additional financial and environmental costs

of coblation technique, our findings suggest that carefully designed large studies are justified to clarify the evidence base around optimal tonsillectomy technique.

Article word count: 2,890 (including tables, figure legends, headings, excluding abstract, key points, and references)

Supporting information:

S1 STROBE_checklist_cross-sectional.pdf

References:

1. Metcalfe C, Muzaffar J, Daultrey C, Coulson C. Coblation tonsillectomy: a systematic review and descriptive analysis. European Archives of Otorhinolaryngology. 2017. 274(6):2637-2647. PMID: 28315933.

2. Heward E, Rocke J, McNally G, Thompson G, Oladokun D, Timms S, et al. The post-operative tonsillectomy (POPT) study: A multi-centre prospective paediatric cohort study. Clinical Otolaryngology. 2024. 49(2):176-184. PMID: 37915294.

3. Smith and Nephew. Coblation Technology for Adenotonsillectomy. [Internet]. [Accessed 8/2/2024]. Available: https://www.smith-nephew.com/en-gb/health-care-professionals/products/ear-nose-and-throat/coblation-technology-for-adenotonsillectomy#overview

4. Roje Z, Racić G, Dogas Z, Pisac VP, Timms M. Postoperative morbidity and histopathologic characteristics of tonsillar tissue following coblation tonsillectomy in children: a prospective randomized single-blind study. Collegium Antropologicum. 2009. 33(1):293-8. PMID: 19408640.

5. Wilson JA, O'Hara J, Fouweather T, Homer T, Stocken DD, Vale L, et al. Conservative management versus tonsillectomy in adults with recurrent acute tonsillitis in the UK (NATTINA): a multicentre, open-label, randomised controlled trial. Lancet. 2023. 17;401(10393):2051-2059. PMID: 37209706.

6. The Royal College of Surgeons. National Prospective Tonsillectomy Audit. 2005. [Accessed March 2024]. Available: https://www.rcseng.ac.uk/-/media/Files/RCS/Library-and-publications/Non-journal-publications/National-Prospective-Tonsillectomy-Audit-Final-Report-2005.pdf

7. Pynnonen M, Brinkmeier JV, Thorne MC, Chong LY, Burton MJ. Coblation versus other surgical techniques for tonsillectomy. Cochrane Database of Systematic Reviews. 2017. 22;8(8):CD004619. PMID: 28828761.

8. Daskalakis D, Tsetsos N, Karagergou S, Goudakos J, Markou K, Karkos P. Intracapsular coblation tonsillectomy versus extracapsular coblation tonsillectomy: a systematic review and a meta-analysis. European Archives of Otorhinolaryngology. 2021. 278(3):637-644. PMID: 32623507.

9. Amin N, Bhargava E, Prentice JG, Shamil E, Walsh M, Tweedie DJ. Coblation intracapsular tonsillectomy in children: A prospective study of 1257 consecutive cases with long-term follow-up. Clinical Otolaryngology. 2021. 46(6):1184-1192. PMID: 33908194.

10. Powell S, Tweedie DJ, Jonas NE, Bateman ND, Keltie K, Sims AJ. Coblation intracapsular tonsillectomy: A cohort study of NHS practice in England using Hospital Episode Statistics. Clinical

Otolaryngology. 2022. 47(3):471-477. Erratum in: Clinical Otolaryngology. 2022. 47(6):707. PMID: 35289094.

11. Marshall A. Ear, Nose and Throat Surgery: GIRFT Programme National Specialty Report. 2019 [Accessed 25 Jan 2024] Available: https://gettingitrightfirsttime.co.uk/wp-content/uploads/2019/10/ENT-Report-Nov19-L-FINAL.pdf

12. NHS Digital. Hospital Admitted Patient Care Activity. [Accessed Jan 2024] Available: https://digital.nhs.uk/data-and-information/publications/statistical/hospital-admitted-patient-care-activity

13. NHS Digital. OPCS-4.9 to OPCS-4.10 Summary of Core Changes. 2022. [Accessed March 2024]. Available: https://digital.nhs.uk/binaries/content/assets/website-assets/isce/0084/0084362022changesummary.pdf

14. Carney AS, Harris PK, MacFarlane PL, Nasser S, Esterman A. The coblation tonsillectomy learning curve. Otolaryngology–Head Neck Surgery. 2008. 138(2):149–152. PMID: 18241706.

Click here to access/download;Figure;Fig 1.png

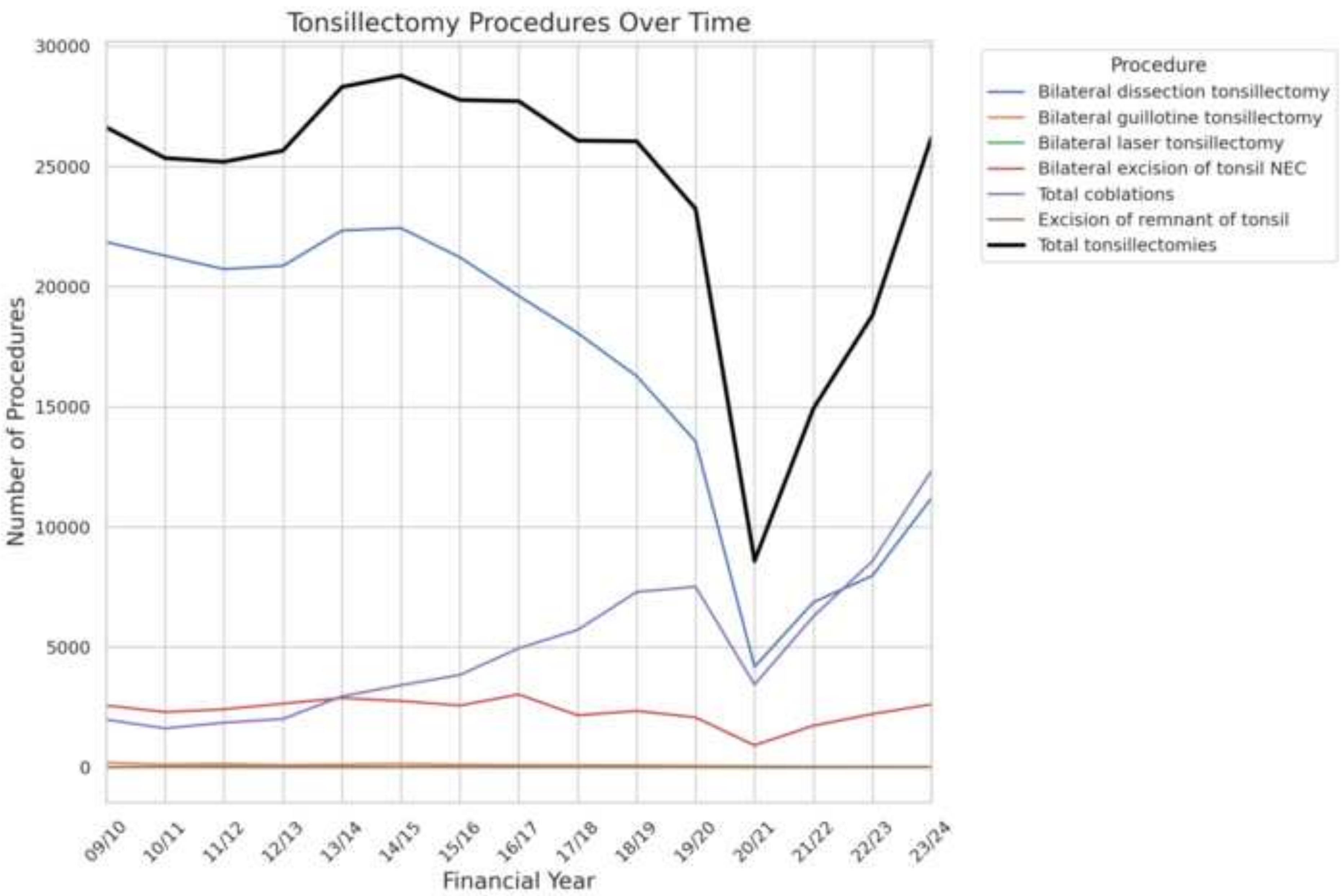

Click here to access/download;Figure;Fig 2.png

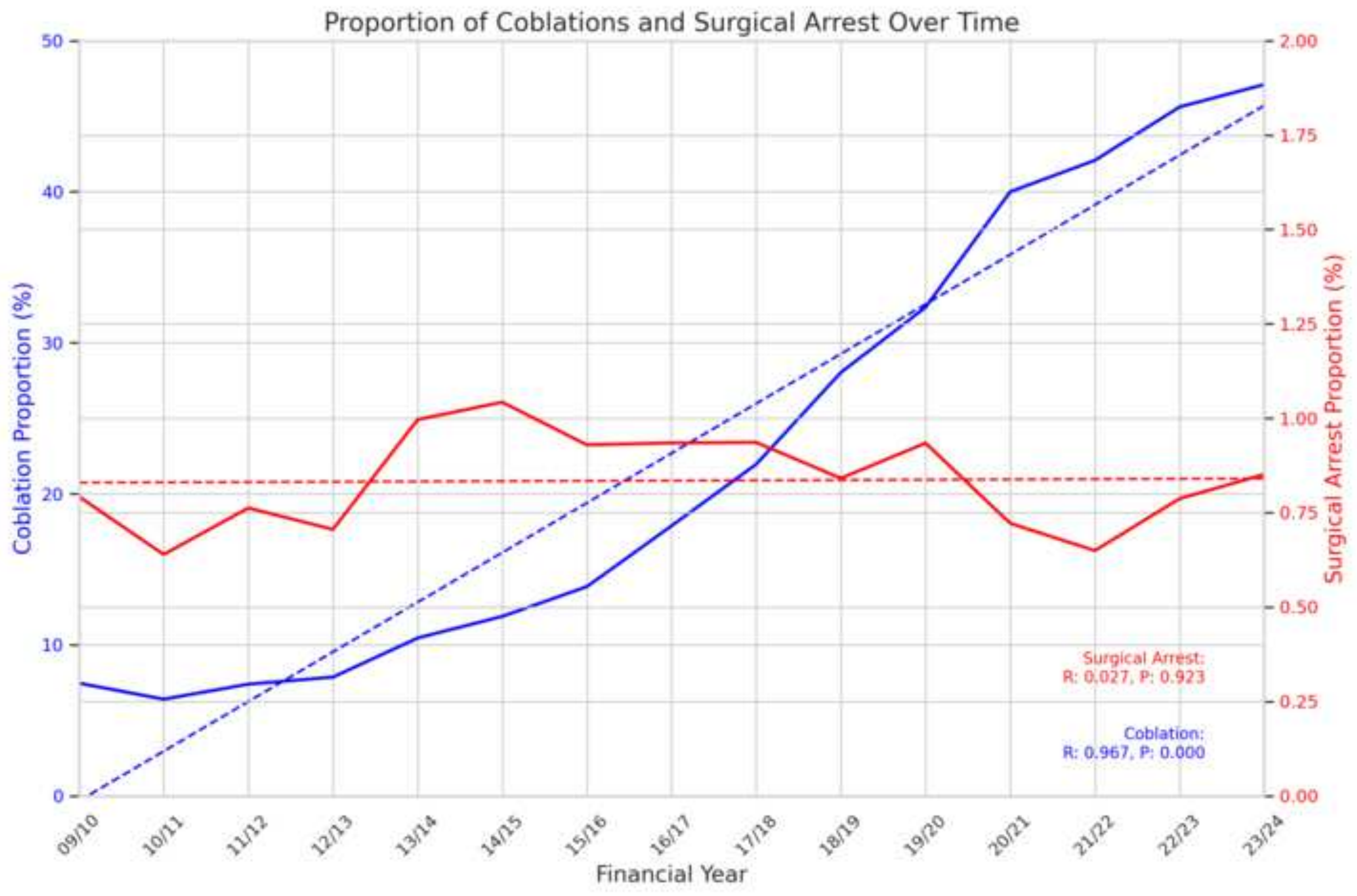

Click here to access/download;Figure;Fig 3.png

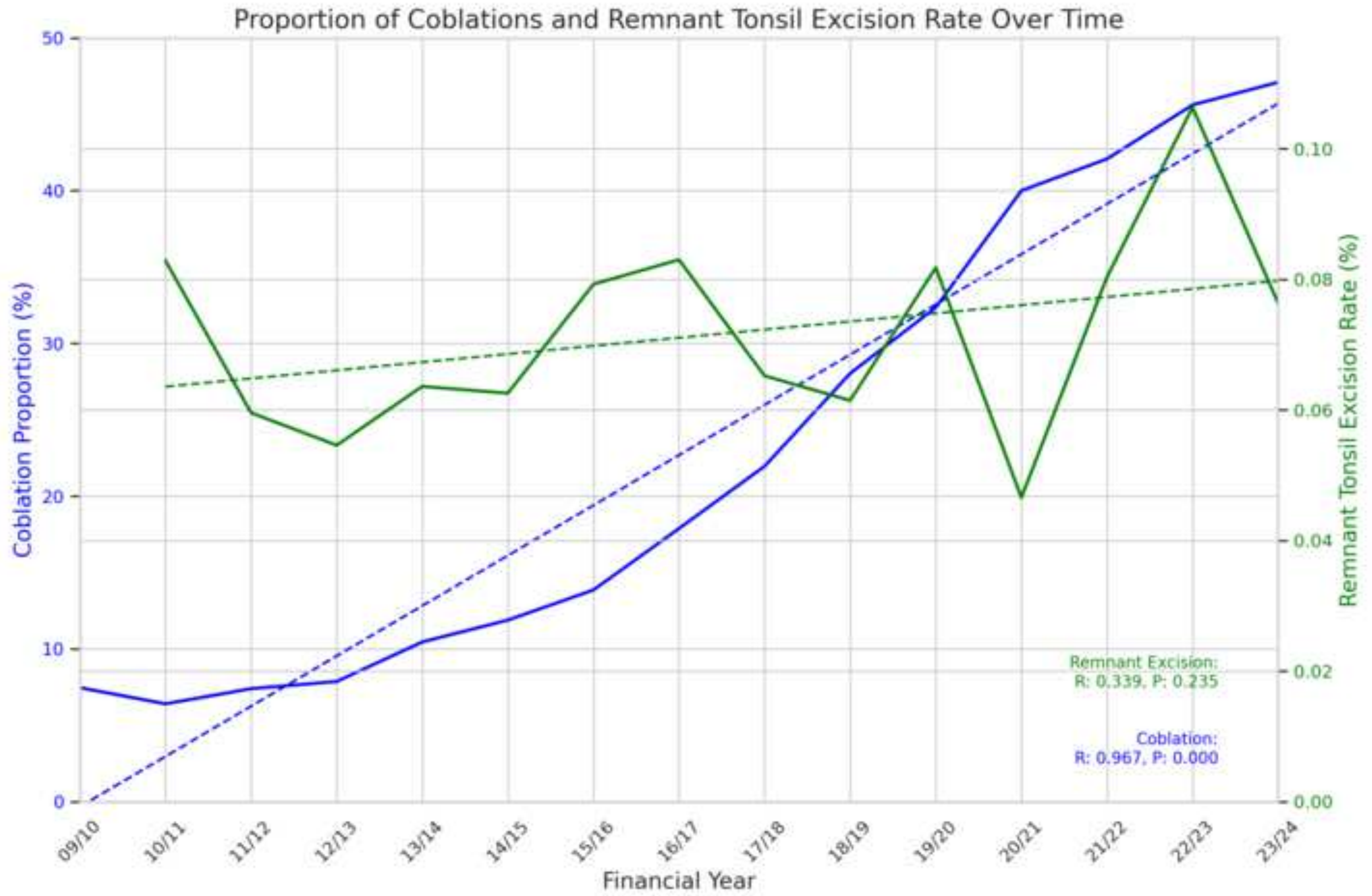

Click here to access/download
Supporting Information
S1 STROBE_checklist_cross-sectional.pdf
