# Supplementary material for: Return to theatre for post-tonsillectomy haemorrhage in children has not fallen with increased use of plasma ablation tonsillectomy: a retrospective analysis of 359,241 tonsillectomies in 15 years of United Kingdom Hospital Episode Statistics
Source: PLoS One. 2025 Jul 28;20(7):e0328251. doi: 10.1371/journal.pone.0328251 (PMC12303344; doi:10.1371/journal.pone.0328251)
Supplement: Attachment — Submitted filename: RESPONSE TO REVIEWERS.docx [file pone.0328251.s004.docx]

**RESPONSE TO REVIEWERS:**

**PONE-D-25-08145**

**Return to theatre for post-tonsillectomy haemorrhage in children has not fallen with increased use of plasma ablation tonsillectomy: a retrospective analysis of 359,241 tonsillectomies in 15 years of United Kingdom Hospital Episode Statistics**

Dear Editor,

Please find the reviewers comments copy and pasted below with our authors response highlighted in red. Please do get in touch if any further clarifications are required.

Yours sincerely,

Mr Jeremy Reid

BMedSc (Hons), MB ChB (Hons), PGDip, FHEA, MRCS (ENT)

Registrar in ENT Surgery

University Hospitals Birmingham NHS Foundation Trust

On behalf of all authors

**3. Have the authors made all data underlying the findings in their manuscript fully available?**

The PLOS Data policy requires authors to make all data underlying the findings described in their manuscript fully available without restriction, with rare exception (please refer to the Data Availability Statement in the manuscript PDF file). The data should be provided as part of the manuscript or its supporting information, or deposited to a public repository. For example, in addition to summary statistics, the data points behind means, medians and variance measures should be available. If there are restrictions on publicly sharing data—e.g. participant privacy or use of data from a third party—those must be specified.

**Reviewer #1:** Yes

**Reviewer #2:** No

**Reviewer #3:** Yes

In response to reviewer 2, all data is freely available from the NHS Digital website, as explained in the manuscript (lines 115-118, lines 154-156) and indicated by reference 12.

**5. Review Comments to the Author**

Please use the space provided to explain your answers to the questions above. You may also include additional comments for the author, including concerns about dual publication, research ethics, or publication ethics. (Please upload your review as an attachment if it exceeds 20,000 characters)

**Reviewer #1:** Excellent review about the most common surgery performed in ORL practice.
The article poses a lot of new points with a fresh conclusion contrary to conventional beliefs.
It just needs one clarification which has been highlighted in the PDF.

Thank you for the encouraging feedback. In response to the comment ‘How will it increase??’ tagged to line 138 of the PDF, the hypothesis is that remnant tonsil procedures may increase due to incomplete excision of tonsillar tissue which can then regrow.

**Reviewer #2:** The authors appear unable to reliably distinguish between intracapsular and extracapsular techniques in the dataset and instead rely on whether plasma ablation was used. To indirectly explore the difference, they introduced a secondary hypothesis, presuming that the intracapsular technique might be associated with increased regrowth and therefore a higher incidence of secondary tonsillectomy. However, this rationale is not clearly articulated in the manuscript, and may be difficult for readers without domain-specific familiarity to follow. A more explicit explanation of this assumption and its relevance to the analysis would be helpful, particularly in the section spanning lines 210–218.

The dataset limitation of being unable to distinguish between intra and extra-capsular use of coblation has not influenced our hypotheses. Therefore, the reason the ‘rationale is not clearly articulated’ is because it is not the rationale for the second hypothesis. The two hypotheses are independent. Regarding this dataset limitation, we do address it at length in the manuscript and discuss its implications for how we understand the data.

Lines 210–218: It is unclear whether microdebrider intracapsular tonsillectomy is currently performed within the NHS. The methodology used in this study appears to assume that it is not, which may warrant clarification. In my own clinical practice, the microdebrider is my preferred technique for intracapsular tonsillectomy over coblation, and I know it is also favored by many of my colleagues in the United States. In my experience, it offers faster operative times and improved visualization of tissue planes near the capsule. Estimated blood loss is typically between 5–10 mL, which is well tolerated, and the minimal use of suction bovie cautery results in low postoperative discomfort. While pain perception is inherently subjective, my anesthesiology colleagues report that my patients experience less postoperative pain compared to those undergoing coblator intracapsular tonsillectomy, though I recognize this observation is highly subjective may be influenced by multiple variables. More objectively, from an institutional standpoint, the microdebrider is significantly more cost-effective than the coblator, as the blades are considerably less expensive than coblation wands. Furthermore, the single-use component of the microdebrider is smaller, resulting in reduced surgical waste. Given that the authors briefly mention the financial and environmental implications of coblation in their conclusion (line 384), a more detailed discussion of these aspects—especially in the context of growing emphasis on sustainable surgical practices—would enhance the manuscript's relevance and practical applicability.

Thank you for sharing your clinical experience however we can only analyse the objective data within our dataset. Hospital Episode Statistics do not include a code to record use of a microdebrider. The authors’ clinical experience is that use of a microdebrider for tonsillectomy would be exceptionally uncommon in UK practice. Furthermore, codes for the 2023/2024 dataset confirm that ‘non-plasma ablation intracapsular procedures’, within which microdebrider tonsillectomy would fall, have minimal contribution to the total number of tonsillectomies, as discussed in lines 211-214.

Line 287. Clarification is needed on how the authors determined that 33% of procedures were performed using the intracapsular technique. Given the acknowledged limitations in identifying surgical approach from coding data, additional detail on the methodology used to arrive at this estimate.

Please refer to lines 207 to 217 where we explain new codes are introduced for the 2023/24 dataset: 4080 (F35.2 Bilateral intracapsular coblation tonsillectomy)/12338 (total number of plasma ablation tonsillectomies for that year) *100 = 33.1%, but is likely more due to coding errors

Line 291. While concerns about regrowth following intracapsular tonsillectomy do exist, there is a paucity of generalizable data to substantiate a meaningful rate of recurrence. Anecdotally, among colleagues with substantial experience using the intracapsular approach, the incidence of regrowth necessitating revision tonsillectomy is reported to be around 1% or lower. Such low recurrence rates, in conjunction with the relatively recent and gradual adoption of the intracapsular technique, may account for the lack of a statistically significant trend in remnant tonsil excision observed in the present analysis in addition to coding inaccuracies that the authors acknowledge.

Line 291 discusses rebleed rate, not regrowth rate. However, reviewer 2’s comments highlight why the second hypothesis was included in this study.

**Reviewer #3:** Authors evaluated the impact of plasma ablation tonsillectomy on the incidence of post-operative haemorrhage requiring surgical intervention in 359,241 children between 2009 and 2014. The use of plasma ablation for tonsillectomy rose; however, there was no change in trend in the rate of return to theatre for control of post-tonsillectomy haemorrhage. Authors concluded that plasma ablation tonsillectomy does not have a superior safety profile with regard to post-operative haemorrhage.

The major strength of the study is the large cohort of patients who had undergone tonsillectomy with multiple techniques.
The major methodological issue is the lack of differentiation between intracapsular and extracapsular plasma ablation tonsillectomy. The study has no information regarding the number of intracapsular tonsillectomies performed prior to 2023. Intracapsular tonsillectomy codes were not available during the entire study period, although, the intracapsular tonsillectomy technique has been practiced during the study period.As authors noted, post-tonsillectomy haemorrhage after intracapsular tonsillectomy technique has been reported with lower post-tonsillectomy bleeding rates. The haemorrhage rate for intracapsular tonsillectomy should be reported separately for intracapsular tonsillectomy codes (F35.1, F35.2).

Specific comments
A)Abstract: Please include study period Added in
B)Methods
1- The study encompassed 15 years; however, the study period should be reported.
dates have not been Please clearly state the study period: The study years are clearly stated in line 157, as well as table 1 (2009/10-2023/24)
2- Please include the description of “Excision of remnant tonsil (F34.5)”. is F34.5 used for regrowth after intracapsular tonsillectomy. The authors have not created these codes, they are presented by NHS Digital. The meaning of a remnant is a small left over after removal of a greater part. Therefore yes, code F34.5 ‘Excision of remnant tonsil’ would refer to regrowth after a previous tonsillectomy.
3- Is it possible to extract data on primary and secondary hemorrhage? Unfortunately not – the analysis is confined to the data which is made public by NHS Digital and they present only a single code F36.5 for Surgical arrest of postoperative bleeding from tonsillar bed

Results:
1-Please report the overall rate of post-tonsillectomy hemorrhage in the entire group The overall rate of return to theatre is described in lines 233-236. We do not think it is helpful to mean the entire study period. The rate for each year is presented in table 1 allowing a reader to calculate a mean if they wanted to.

2-Please report the post-tonsillectomy hemorrhage rate for confirmed intracapsular tonsillectomy codes (F35.1, F35.2). This is not possible – NHS Digital share the total number of each procedure (i.e., each OPCS code) in a year – the data is not paired
