# Supplementary material for: Return to theatre for post-tonsillectomy haemorrhage in children has not fallen with increased use of plasma ablation tonsillectomy: a retrospective analysis of 359,241 tonsillectomies in 15 years of United Kingdom Hospital Episode Statistics
Source: PLoS One. 2025 Jul 28;20(7):e0328251. doi: 10.1371/journal.pone.0328251 (PMC12303344; doi:10.1371/journal.pone.0328251)
Supplement: Peer review history

# Peer review: Decision Letter 0

**DOI:** 10.1371/journal.pone.0328251.r001

**Authors:** Steve Zimmerman

Dear Dr. Reid,

Thank you for submitting your manuscript to PLOS ONE. After careful consideration, we feel that it has merit but does not fully meet PLOS ONE’s publication criteria as it currently stands. Therefore, we invite you to submit a revised version of the manuscript that addresses the points raised during the review process.

The manuscript has been evaluated by three reviewers, and their comments are available below.

Please submit your revised manuscript by May 30 2025 11:59PM. If you will need more time than this to complete your revisions, please reply to this message or contact the journal office at plosone@plos.org . When you're ready to submit your revision, log on to https://www.editorialmanager.com/pone/ and select the 'Submissions Needing Revision' folder to locate your manuscript file.

- A rebuttal letter that responds to each point raised by the academic editor and reviewer(s). You should upload this letter as a separate file labeled 'Response to Reviewers'.
- A marked-up copy of your manuscript that highlights changes made to the original version. You should upload this as a separate file labeled 'Revised Manuscript with Track Changes'.
- An unmarked version of your revised paper without tracked changes. You should upload this as a separate file labeled 'Manuscript'.

If you would like to make changes to your financial disclosure, please include your updated statement in your cover letter. Guidelines for resubmitting your figure files are available below the reviewer comments at the end of this letter.

If applicable, we recommend that you deposit your laboratory protocols in protocols.io to enhance the reproducibility of your results. Protocols.io assigns your protocol its own identifier (DOI) so that it can be cited independently in the future. For instructions see: https://journals.plos.org/plosone/s/submission-guidelines#loc-laboratory-protocols . Additionally, PLOS ONE offers an option for publishing peer-reviewed Lab Protocol articles, which describe protocols hosted on protocols.io. Read more information on sharing protocols at https://plos.org/protocols?utm_medium=editorial-email&utm_source=authorletters&utm_campaign=protocols .

We look forward to receiving your revised manuscript.

Kind regards,

Steve Zimmerman, PhD

Senior Editor, PLOS One

Journal Requirements:

When submitting your revision, we need you to address these additional requirements.

1. Please ensure that your manuscript meets PLOS ONE's style requirements, including those for file naming. The PLOS ONE style templates can be found at 

https://journals.plos.org/plosone/s/file?id=wjVg/PLOSOne_formatting_sample_main_body.pdf and 

https://journals.plos.org/plosone/s/file?id=ba62/PLOSOne_formatting_sample_title_authors_affiliations.pdf

[Note: HTML markup is below. Please do not edit.]

Reviewers' comments:

Reviewer's Responses to Questions

Comments to the Author

1. Is the manuscript technically sound, and do the data support the conclusions?

Reviewer #1: Yes

Reviewer #2: Yes

Reviewer #3: Yes

**********

2. Has the statistical analysis been performed appropriately and rigorously?

Reviewer #1: Yes

Reviewer #2: Yes

Reviewer #3: Yes

**********

3. Have the authors made all data underlying the findings in their manuscript fully available?

The PLOS Data policy

Reviewer #1: Yes

Reviewer #2: No

Reviewer #3: Yes

**********

4. Is the manuscript presented in an intelligible fashion and written in standard English?

Reviewer #1: Yes

Reviewer #2: Yes

Reviewer #3: Yes

**********

Reviewer #1: Excellent review about the most common surgery performed in ORL practice.

The article poses a lot of new points with a fresh conclusion contrary to conventional beliefs.

It just needs one clarification which has been highlighted in the PDF.

Reviewer #2: The authors appear unable to reliably distinguish between intracapsular and extracapsular techniques in the dataset and instead rely on whether plasma ablation was used. To indirectly explore the difference, they introduced a secondary hypothesis, presuming that the intracapsular technique might be associated with increased regrowth and therefore a higher incidence of secondary tonsillectomy. However, this rationale is not clearly articulated in the manuscript, and may be difficult for readers without domain-specific familiarity to follow. A more explicit explanation of this assumption and its relevance to the analysis would be helpful, particularly in the section spanning lines 210–218.

Lines 210–218: It is unclear whether microdebrider intracapsular tonsillectomy is currently performed within the NHS. The methodology used in this study appears to assume that it is not, which may warrant clarification. In my own clinical practice, the microdebrider is my preferred technique for intracapsular tonsillectomy over coblation, and I know it is also favored by many of my colleagues in the United States. In my experience, it offers faster operative times and improved visualization of tissue planes near the capsule. Estimated blood loss is typically between 5–10 mL, which is well tolerated, and the minimal use of suction bovie cautery results in low postoperative discomfort. While pain perception is inherently subjective, my anesthesiology colleagues report that my patients experience less postoperative pain compared to those undergoing coblator intracapsular tonsillectomy, though I recognize this observation is highly subjective may be influenced by multiple variables. More objectively, from an institutional standpoint, the microdebrider is significantly more cost-effective than the coblator, as the blades are considerably less expensive than coblation wands. Furthermore, the single-use component of the microdebrider is smaller, resulting in reduced surgical waste. Given that the authors briefly mention the financial and environmental implications of coblation in their conclusion (line 384), a more detailed discussion of these aspects—especially in the context of growing emphasis on sustainable surgical practices—would enhance the manuscript's relevance and practical applicability.

Line 287. Clarification is needed on how the authors determined that 33% of procedures were performed using the intracapsular technique. Given the acknowledged limitations in identifying surgical approach from coding data, additional detail on the methodology used to arrive at this estimate.

Line 291. While concerns about regrowth following intracapsular tonsillectomy do exist, there is a paucity of generalizable data to substantiate a meaningful rate of recurrence. Anecdotally, among colleagues with substantial experience using the intracapsular approach, the incidence of regrowth necessitating revision tonsillectomy is reported to be around 1% or lower. Such low recurrence rates, in conjunction with the relatively recent and gradual adoption of the intracapsular technique, may account for the lack of a statistically significant trend in remnant tonsil excision observed in the present analysis in addition to coding inaccuracies that the authors acknowledge.

Reviewer #3: Authors evaluated the impact of plasma ablation tonsillectomy on the incidence of post-operative haemorrhage requiring surgical intervention in 359,241 children between 2009 and 2014. The use of plasma ablation for tonsillectomy rose; however, there was no change in trend in the rate of return to theatre for control of post-tonsillectomy haemorrhage. Authors concluded that plasma ablation tonsillectomy does not have a superior safety profile with regard to post-operative haemorrhage.

The major strength of the study is the large cohort of patients who had undergone tonsillectomy with multiple techniques.

The major methodological issue is the lack of differentiation between intracapsular and extracapsular plasma ablation tonsillectomy. The study has no information regarding the number of intracapsular tonsillectomies performed prior to 2023. Intracapsular tonsillectomy codes were not available during the entire study period, although, the intracapsular tonsillectomy technique has been practiced during the study period.As authors noted, post-tonsillectomy haemorrhage after intracapsular tonsillectomy technique has been reported with lower post-tonsillectomy bleeding rates. The haemorrhage rate for intracapsular tonsillectomy should be reported separately for intracapsular tonsillectomy codes (F35.1, F35.2).

Specific comments

A)Abstract: Please include study period

B)Methods

1- The study encompassed 15 years; however, the study period should be reported.

dates have not been Please clearly state the study period:

2- Please include the description of “Excision of remnant tonsil (F34.5)”. is F34.5 used for regrowth after intracapsular tonsillectomy.

3- Is it possible to extract data on primary and secondary hemorrhage?

Results:

1-Please report the overall rate of post-tonsillectomy hemorrhage in the entire group

2-Please report the post-tonsillectomy hemorrhage rate for confirmed intracapsular tonsillectomy codes (F35.1, F35.2).

**********

what does this mean? ). If published, this will include your full peer review and any attached files.

If you choose “no”, your identity will remain anonymous but your review may still be made public.

**Do you want your identity to be public for this peer review?** For information about this choice, including consent withdrawal, please see our Privacy Policy

Reviewer #1: **Yes: ** Saai Ram Thejas

Reviewer #2: No

Reviewer #3: No

**********

[NOTE: If reviewer comments were submitted as an attachment file, they will be attached to this email and accessible via the submission site. Please log into your account, locate the manuscript record, and check for the action link "View Attachments". If this link does not appear, there are no attachment files.]

While revising your submission, please upload your figure files to the Preflight Analysis and Conversion Engine (PACE) digital diagnostic tool, https://pacev2.apexcovantage.com/ . PACE helps ensure that figures meet PLOS requirements. To use PACE, you must first register as a user. Registration is free. Then, login and navigate to the UPLOAD tab, where you will find detailed instructions on how to use the tool. If you encounter any issues or have any questions when using PACE, please email PLOS at figures@plos.org . Please note that Supporting Information files do not need this step.

Attachment Submitted filename: PONE-D-25-08145.pdf

# Author comment: Author response to Decision Letter 1

**DOI:** 10.1371/journal.pone.0328251.r002

30 May 2025

Please see a point by point response in the attached documents. With kind regards.

Attachment Submitted filename: RESPONSE TO REVIEWERS.docx

# Peer review: Decision Letter 1

**DOI:** 10.1371/journal.pone.0328251.r003

**Authors:** Randall J. Kimple

Return to theatre for post-tonsillectomy haemorrhage in children has not fallen with increased use of plasma ablation tonsillectomy: a retrospective analysis of 359,241 tonsillectomies in 15 years of United Kingdom Hospital Episode Statistics

PONE-D-25-08145R1

Dear Dr. Reid,

We’re pleased to inform you that your manuscript has been judged scientifically suitable for publication and will be formally accepted for publication once it meets all outstanding technical requirements.

Within one week, you’ll receive an e-mail detailing the required amendments. When these have been addressed, you’ll receive a formal acceptance letter and your manuscript will be scheduled for publication.

An invoice will be generated when your article is formally accepted. Please note, if your institution has a publishing partnership with PLOS and your article meets the relevant criteria, all or part of your publication costs will be covered. Please make sure your user information is up-to-date by logging into Editorial Manager at Editorial Manager®  and clicking the ‘Update My Information' link at the top of the page. If you have any questions relating to publication charges, please contact our Author Billing department directly at authorbilling@plos.org.

If your institution or institutions have a press office, please notify them about your upcoming paper to help maximize its impact. If they’ll be preparing press materials, please inform our press team as soon as possible -- no later than 48 hours after receiving the formal acceptance. Your manuscript will remain under strict press embargo until 2 pm Eastern Time on the date of publication. For more information, please contact onepress@plos.org.

Kind regards,

Randall J. Kimple

Academic Editor

PLOS ONE

Additional Editor Comments (optional):

Reviewers' comments:

Reviewer's Responses to Questions

Comments to the Author

Reviewer #1: All comments have been addressed

Reviewer #2: All comments have been addressed

Reviewer #4: (No Response)

**********

2. Is the manuscript technically sound, and do the data support the conclusions?

Reviewer #1: Partly

Reviewer #2: Yes

Reviewer #4: Yes

**********

3. Has the statistical analysis been performed appropriately and rigorously?

Reviewer #1: Yes

Reviewer #2: Yes

Reviewer #4: Yes

**********

4. Have the authors made all data underlying the findings in their manuscript fully available?

The PLOS Data policy

Reviewer #1: Yes

Reviewer #2: Yes

Reviewer #4: Yes

**********

5. Is the manuscript presented in an intelligible fashion and written in standard English?

Reviewer #1: Yes

Reviewer #2: Yes

Reviewer #4: Yes

**********

Reviewer #1: Respected Authors,

This is particularly useful for all Otorhinolaryngologists because the amount of pressure associated with a post-tonsillectomy bleed is immense.

Good job with the article.

Regards

Reviewer #2: Thank you for your responses to the reviewer comments. I am satisfied with the revisions and have no further concerns or suggestions at this time.

Reviewer #4: The study authors have presented a 15-year retrospective analysis with data from the UK HES on the incidence of return to theatre for post-tonsillectomy haemorrhage in children. They have shown that with increasing use of plasma ablation tonsillectomy this incidence has not fallen. They have also demonstrated that with increasing use of plasma ablation tonsillectomy a non statistically significant upward trend of excision of remnant tonsil.

In reading the prior reviewers comments, the study authors have addressed all points satisfactorily and I have no further recommendations.

**********

what does this mean? ). If published, this will include your full peer review and any attached files.

If you choose “no”, your identity will remain anonymous but your review may still be made public.

**Do you want your identity to be public for this peer review?** For information about this choice, including consent withdrawal, please see our Privacy Policy

Reviewer #1: **Yes: ** Saai Ram Thejas

Reviewer #2: No

Reviewer #4: No

**********

# Editor report: Acceptance letter

**DOI:** 10.1371/journal.pone.0328251.r004

**Authors:** Randall J. Kimple

PONE-D-25-08145R1

PLOS ONE

Dear Dr. Reid,

I'm pleased to inform you that your manuscript has been deemed suitable for publication in PLOS ONE. Congratulations! Your manuscript is now being handed over to our production team.

At this stage, our production department will prepare your paper for publication. This includes ensuring the following:

* All references, tables, and figures are properly cited

* All relevant supporting information is included in the manuscript submission,

* There are no issues that prevent the paper from being properly typeset

You will receive further instructions from the production team, including instructions on how to review your proof when it is ready. Please keep in mind that we are working through a large volume of accepted articles, so please give us a few days to review your paper and let you know the next and final steps.

Lastly, if your institution or institutions have a press office, please let them know about your upcoming paper now to help maximize its impact. If they'll be preparing press materials, please inform our press team within the next 48 hours. Your manuscript will remain under strict press embargo until 2 pm Eastern Time on the date of publication. For more information, please contact onepress@plos.org.

If we can help with anything else, please email us at customercare@plos.org.

Thank you for submitting your work to PLOS ONE and supporting open access.

Kind regards,

PLOS ONE Editorial Office Staff

on behalf of

Dr. Randall J. Kimple

Academic Editor

PLOS ONE
